# Is one additional phone call enough? - Effectiveness of additional human support to reduce dropout from an internet-based intervention for depressive symptoms: A randomized-controlled trial

**DOI:** 10.1016/j.invent.2025.100818

**Published:** 2025-03-11

**Authors:** Sophie Christine Eicher, Manuel Heinrich, Pavle Zagorscak, Annette Brose, Christine Knaevelsrud

**Affiliations:** Division of Clinical Psychological Intervention, Department of Education and Psychology, Freie Universität Berlin, Germany

**Keywords:** Internet-based intervention, Depression, Treatment dropout, Human support

## Abstract

**Background:**

Internet-Based Interventions (IBIs) are effective treatments for mental disorders, but their implementation faces challenges, particularly in addressing high dropout rates. Adding more human support or guidance might reduce treatment dropout rates in IBIs, but it may also limit scalability. Therefore, small, easy-to-implement, guidance-based add-on interventions are warranted to reduce dropout rates. This study tests if offering one additional brief phone contact reduces treatment dropout rates in an IBI for depressive symptoms with written guidance.

**Methods:**

We analyze data from *N* = 394 individuals participating in an IBI for depression. The intervention comprises seven CBT-based modules with module-wise written semi-standardized feedback from psychotherapists (guided IBI). Previous research applying the same IBI in adults with self-reported symptoms of depression found increased dropout rates after the second module. In the study group, therapists offered an additional brief phone call after the second module (*n* = 206). In the control group, no additional phone calls were offered (*n* = 188). We present descriptive statistics regarding the intervention course for both groups. We conducted a logistic regression to examine the preventive effect of the additional phone call on treatment dropout.

**Results:**

Pooled dropout rates in the study group were 30.5 % (*n* = 63), and in the control group 36.1 % (*n* = 68), with a risk difference of about 6 % points favoring the study group. The odds ratios ranged from 1.25 to 1.33, and the relative risks ranged from 1.08 to 1.10. However, all confidence intervals overlap zero, indicating that all effect estimates are statistically non-significant.

**Conclusion:**

We tested a strategy of additional human contact to reduce treatment dropout rates in a guided IBI for depressive symptoms. All estimates descriptively favored the study group, but were small and non-significant. Further research is needed to determine how additional contact can be employed to reduce treatment dropout.

## Highlights

• Offering additional phone contact was tested to reduce treatment dropout rates.
• Results suggest additional phone contact may help, but no significant effect found.
• Identifying who benefits is key for reducing treatment dropout rates effectively.
• Additional contact shows promise, but how to best leverage it is unclear.

## 1 Introduction

While internet-based interventions (IBIs) for depression are effective (Karyotaki et al., 2018), many individuals do not complete these interventions. Torous et al. (2020) reported a publication-bias-corrected dropout rate of 48 % in a meta-analysis of smartphone-delivered interventions. Similarly, Moshe et al. (2021) found that nearly half of the participants in IBIs for depressive symptoms dropped out early. Although different studies use different approaches to operationalize dropout, treatment dropout typically refers to patients who stop using the intervention before they have been exposed to all the intervention material (Musiat et al., 2022). Therefore, individuals dropping out early are at risk of not experiencing the full benefit of an IBI (Karyotaki et al., 2017), and the full potential of IBIs might not be fully met. Thus, it is of great importance to understand how we can prevent people from dropping out.

The factor most consistently reported to benefit adherence is therapeutic guidance, defined as the level of human/therapeutic support given throughout the IBI (Torous et al., 2020). Guidance can vary in various ways (synchronous vs. asynchronous; email contact vs. phone contact). In IBIs for depressive symptoms, guidance usually consists of asynchronous written feedback (Moshe et al., 2021). Comparative studies on IBIs with and without guidance revealed that guidance is consistently associated with reduced dropout rates (Musiat et al., 2022). These findings raise the question of whether additional phone-based guidance can be leveraged to further reduce dropout rates. This seems particularly promising, given that dropout is potentially more likely the more *virtual* the support is (Eysenbach, 2005). In more detail, synchronous telephone contact is less virtual than contact via email, it is more personal, less prone to misunderstandings, and more interactive. These aspects seem important against the background that even individuals participating in guided interventions would prefer more personal contact (Fenski et al., 2021).

Evidence on the benefit of additional phone support in guided interventions is scarce. In a pragmatic randomized controlled trial, Pihlaja et al. (2020) report that additional weekly telephone support reduced treatment dropout from an IBI with otherwise written guidance. Besides, several studies examined the effect of additional phone support in *unguided* interventions (e.g. Andersson et al., 2003; Gilbody et al., 2017; Kenwright et al., 2005; Mira et al., 2017). Although some studies failed to reach statistical significance, they all report descriptively reduced dropout rates in the intervention group.

All studies reviewed above have in common that they provided phone contacts regularly throughout the treatment (e.g., weekly). Despite the fact that adding more guidance throughout the intervention could reduce dropout rates, it has to be considered, however, that it might have a negative impact on scalability. For example, Pihlaja et al. (2020) reported *M* = 73 min of additional telephone contact for patients with depressive symptoms. Therefore, such resource allocation should be carefully considered and weighed against the interventions' effects.

On this background – generally positive effects of phone support in IBIs and related disadvantages in view of resources – this study aimed to find out whether a small, easy-to-implement intervention such as a short, one-off telephone call can reduce dropout rates in interventions that already provide written guidance. The general idea behind the study is that we aim to avoid negative effects on scalability while capitalizing on benefits (synchronicity, person-specificity) by implementing a single phone call toward the beginning of an IBI. IBIs are typically short (6 to 12 weeks); thus, a single contact might already improve the participant's intervention experience. Also, the focus on a single phone call aligns with other studies that have discussed to optimize therapists' time efficiently in IBI settings (e.g. Hadjistavropoulos et al., 2017; Hadjistavropoulos et al., 2019).

Regarding the way a single telephone call may prevent dropout, we see two possibilities. First, it might function as a motivation to continue *until* the phone call occurs. Second, the counselor may motivate treatment continuation by educating about potential barriers in the following treatment phases, which may prevent dropout after the phone-call. The provided phone call in this study had the explicit purpose of providing support in case of any difficulties in working through the IBI and should motivate to continue the treatment. Due to the lack of well-validated criteria for identifying individuals at increased risk of dropout in an IBI, we opted for a completely randomized allocation of all participants.

In summary, our objective is to test if offering only one additional brief phone contact reduces treatment dropout in an IBI for depressive symptoms with written guidance.

## 2 Methods

### 2.1 Sample

**Fig. 1:**
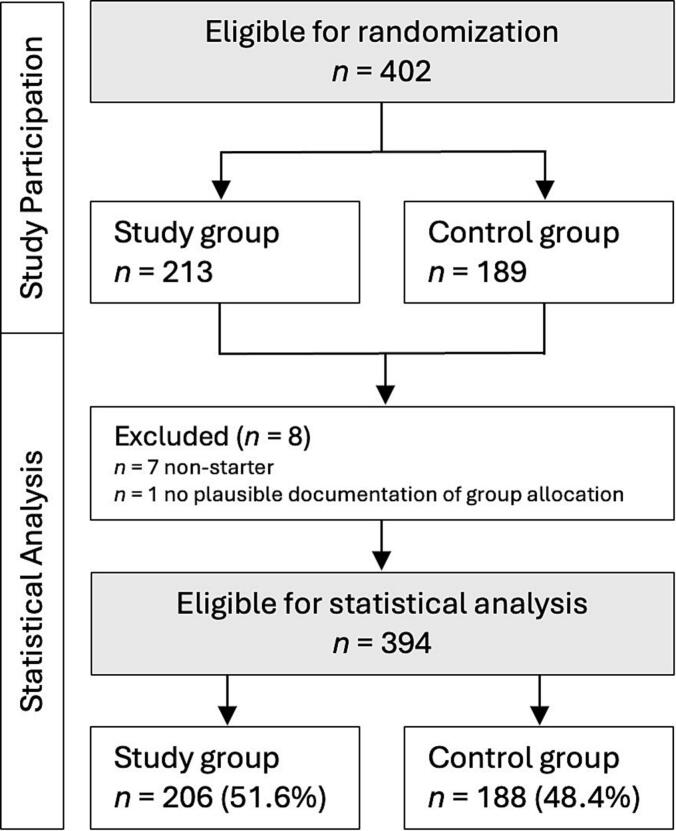
Flowchart study participants.

We analyzed data collected from individuals insured by a large German health insurance company (*>1 million insurants*) using an IBI for depression (TK-DepressionsCoach; TKDC) between February 2023 and May 2023. Interested persons self-registered for the IBI via an Internet platform. First, participants completed an online screening procedure. Participants were excluded if they reported too mild (BDI-II < 14) or too severe symptoms (PHQ-9 > 20) of depression or elevated levels of suicidal thought (PHQ-9 or BDI-II item 9 score ≥ 2). Secondly, participants participated in a structured clinical interview (SCID-I, section A on affective disorders, and section B on psychotic symptoms; Wittchen et al., 1997) conducted by trained interviewers via telephone. Interviewers excluded participants reporting lifetime symptoms of mania, psychosis, or elevated levels of suicidality. Participants meeting the inclusion criteria were randomly assigned (1:1 allocation ratio) to two groups (study group, control group, see Fig. 1).

Given the nature of the study, participants knew whether they were in the study or control group, and the therapists were also aware of the assigned group. The Research Ethics Committee of Freie Universität Berlin approved the study (Nr. 070.2013, and amendments 125.2016, 004.2020, and 052.2022), and participants gave informed consent.

### 2.2 Description of the IBI

All randomized participants received access to the same treatment material. The IBI is a CBT-based program aiming to reduce depressive symptoms and strengthen self-management abilities. The IBI consists of seven consecutive modules (M1-M7). The intervention starts with two expressive writing tasks (M1, M2), followed by behavioral activation (M3-M4), cognitive restructuring (M5-M6), and a final writing task focusing on relapse prevention (M7). All participants received semi-standardized feedback from counselors (trained psychotherapists) via written messages after each completed module. Furthermore, counselors provided contact on demand in case of symptom deterioration or technical problems. Earlier studies demonstrate the interventions' effectiveness (Brose et al., 2023; Heinrich et al., 2025; Zagorscak et al., 2018).

### 2.3 Study group (Strategy to prevent dropout)

The counselors informed individuals assigned to the study group in a written welcome message that they would receive an additional telephone call after completing the second module (i.e. after participants had written the second letter). The counselors asked participants to send a message containing three available time slots in their feedback on the first writing task. Counselors were instructed to spend about 15 min per call and received a detailed guideline outlining the structure of the phone calls (Supplementary material).

Previous research has pinpointed the highest number of users dropping out of the IBI during the first two modules (Brose et al., 2023). Therefore, counselors offered a phone call after the second module. The phone call had two sections. First, counselors discussed the participants' previous experience with the IBI and the treatment goals participants formulated within the second writing task. The motivation was to recap what participants intended to achieve and discuss unrealistic treatment goals. Second, the content of the upcoming treatment module (M3, behavioral activation) was discussed. In the third module, the participants were encouraged to use an activity planner to schedule enjoyable and meaningful activities. Completing tasks related to behavioral activation can be difficult for people with depressive symptoms (Hautzinger, 2021; Martell et al., 2010). Counselors addressed expected difficulties (e.g., exhaustion, passivity, and avoidance) and tried to manage expectations. The conversation guidelines followed recommendations by Hautzinger (2021) and the principles of behavioral activation formulated by Martell et al. (2010). Participants could refuse to receive the phone call. In this case, the counselors would send a standardized message (Supplementary material) that followed the same subjects as the phone call guideline.

### 2.4 Covariates

*Depressive symptoms* were measured with the Beck-Depression-Inventory-II (BDI-II; Hautzinger et al., 2006) and the Patient Health Questionnaire-9 (PHQ-9; Löwe et al., 2003). *Anxiety symptoms* were measured with the Generalized Anxiety Disorder Scale-7 (GAD-7; Spitzer et al., 2006). *Feelings of stress* were measured with the Patient Health Questionnaire – stress module (PHQ-stress; Löwe et al., 2003). All instruments have demonstrated good psychometric properties (Spitzer et al., 1999; Terrill et al., 2015; Wang and Gorenstein, 2013; Zhong et al., 2015). Participants completed questionnaires and provided sociodemographic variables at baseline.

### 2.5 Outcome measure

The primary intervention outcome was treatment dropout. Similar to former studies using the same intervention (Brose et al., 2023; Zagorscak et al., 2018), we considered participants “dropped out” if they did not start to work with all seven treatment modules. “Start to work” is operationalized as having gained access to the next module by completing the PHQ-9 that is administered at the beginning of every module. Participants that completed the module-specific PHQ-9 at least saw the psychoeducation of the respective module since it is automatically displayed after questionnaire completion. This operationalization was chosen to maintain consistency across multiple papers using the same intervention (e.g., Brose et al., 2023; Zagorscak et al., 2018), where participants were considered as “dropped out” if they did not start all seven treatment modules.”

### 2.6 Statistical analysis

We conducted all statistical analyses in RStudio (Version 2023.9.0.463). The objective was to test the effectiveness of offering participants an additional phone call to reduce dropout. Treatment effects were estimated using logistic regression models. Following recommendations from Wei et al. (2024), we report unconditional, conditional, and unconditional adjusted effect estimates. In the unconditional model, the binary group indicator is the only regressor. For the conditional effect estimate (e.g., the expected effect given certain covariate values), we considered sex, age, level of education, anxiety, depressive symptoms, and stress as covariates. The same covariates were used to evaluate the adjusted unconditional effect. Here, the estimate is standardized with respect to the covariate distribution. All covariates are known predictors for dropout from IBI (see Karyotaki et al., 2015).

The main analysis followed the intention-to-treat principle among individuals who started the intervention after being randomized. The ITT effect considers (1) that some did not receive the phone call *although* they were interested in receiving it (e.g., due to scheduling issues) and (2) others who did not receive the call because they were not interested. Therefore, the estimates represent the group difference under imperfect adherence. We report effects in terms of odds ratio (e.g., the factor by which the odds of completing the program in the study group is increased) and relative risks (e.g., the factor by which the probability of completing the intervention is increased in the study group).

For the main analysis, we had to consider that the operation of the intervention platform was discontinued on August 31, 2023. Although all participants had ample time to complete the 6 to 8-week intervention program, there were some individuals (7.9 %, *n* = 31) who, due to extended periods of inactivity, could have still completed the program if the platform had run longer. However, since these individuals could no longer log in after the platform's discontinuation, the dropout status was considered censored (i.e., the true dropout status is unknown since we do not know if these individuals would have had the opportunity to complete the program). We imputed censored data under the missing-at-random assumption. We excluded non-starters (*n* = 7, Fig. 1). This exclusion is appropriate, as the randomized individuals learned about their assigned group after they started the IBI. We also excluded *n* = 1 participant with no plausible documentation of group allocation (Fig. 1).

For the analysis, we combined bootstrapping and multiple imputations following the Boot MI approach (von Hippel and Bartlett, 2021). Following the recommendations for handling missing data by Wei et al. (2024) and von Hippel and Bartlett (2021), we bootstrap sampled (B = 1000) the observed data and applied multiple imputations (M = 2, number of iterations = 5) with *mice* 3.0 package (van Buuren and Groothuis-Oudshoorn, 2011) via predictive mean matching to each of these samples. Furthermore, we conducted a comprehensive sensitivity analysis. First, we explored a hypothetical scenario where all interested individuals received the phone call. Therefore, we imputed the dropout status of individuals not receiving the phone call due to scheduling and repeated the analysis. Second, we considered the dropout status of all individuals with censored data as “dropped out” and addressed the risk of over-optimistic imputations under missing-at-random assumptions (MAR). Lastly, we also report the result of a complete-case analysis.

## 3 Results

### 3.1 Sample

Overall, *N* = 394 individuals (98.1 % of all randomized individuals) started the IBI (study group: *n* = 206, control group: *n* = 188). The mean age was *M* = 42.31 years (*SD* = 12.47, range 20 to 75). The majority of participants were female (63.2 %). The mean BDI-II score was *M* = 25.19 (*SD* = 6.66), indicating moderate depressive symptoms. Table 1 summarizes participant characteristics.

**Table 1:** Participant characteristics.

	Entire sample	Study group	Control group
Variables	*N* = 394	*n* = 206	*n* = 188
Age in years (*M, SD*)	42.31, 12.47	42.11, 12.59	42.29, 12.26
Gender *n* (% female)	249 (63.2)	138 (67.0)	111 (59.0)
Education *n* (%)			
No certificate	1 (0.3)	0	1 (0.5)
Lower Secondary	9 (2.3)	3 (1.5)	6 (3.2)
Secondary school	63 (16.0)	31 (15.1)	32 (17.0)
Trade school	87 (22.1)	41 (19.9)	46 (24.5)
College	42 (10.7)	19 (9.2)	23 (12.2)
University	192 (48.7)	112 (54.4)	80 (42.6)
			
*Psychopathological symptoms (M, SD)*			
Depressive symptoms (BDI-II)	25.19, 6.66	24.86, 6.61	25.54, 6.71
Depressive symptoms (PHQ-9)	12.84, 3.58	12.67, 3.59	13.03, 3.58
Anxiety (GAD-7)	10.82, 3.94	10.79, 3.80	10.85, 4.10
Stress (PHQ-stress)	9.39, 3.48	9.17, 3.62	9.63, 3.32

*Note. M* = mean, *SD* = standard deviation; BDI-II = Beck's Depression inventory; GAD-7 = General Anxiety Disorder Screener; PHQ-9 = Patient Health Questionnaire-9; PHQ-stress = Patient Health Questionnaire Stress Module.

### 3.2 Dropout over the intervention course

Table 2 provides a detailed course of dropout rates across the course of the IBI. On average, participants completed *M* = 4.79 modules (study group: *M* = 4.87; control group *M* = 4.7). Dropout occurred most frequently between M1-M2 and M3 (12.1 %, Table 2). Overall, 11.2 % of the participants assigned to the study group and 13.3 % assigned to the control group dropped out before starting M3. Among those who started with M3, 17.5 % (study group) and 17.8 % (control group) dropped out before working with all treatment modules. The increase in dropout rate for each module was descriptively lower in the study group except for M4 (see Table 2).

**Table 2:** Dropout for the different modules.

Group	M1	M3	M4	M5	M6	M7	Finished Modules (*M*, *SD*)
Entire Sample *n* (%) *n* = 363⁎	0	44 (12.1) *+12.1*	64 (17.6) *+5.5*	82 (22.6) *+5.0*	102 (28.1) *+5.5*	120 (33.1) *+5.0*	4.8 (1.8)
Study Group *n* (%) *n* = 197⁎	0	22 (11.2) *+11.2*	35 (17.8) *+6.6*	44 (22.3) *+4.0*	51 (25.9) *+3.6*	59 (29.9) *+4.0*	4.9 (1.8)
Control Group *n* (%) *n* = 166⁎	0	22 (13.3) *+13.3*	29 (17.5) *+4.2*	38 (22.9) *+5.4*	51 (30.7) *+7.8*	61 (36.7) *+6.0*	4.7 (1.8)

⁎ *Note.* censored data excluded n = 31 (study group *n* = 9, control group *n* = 22). *Italic* print represents the increase in dropout rate.

### 3.3 Implementation of additional contact

Overall, *n* = 174 individuals (84.5 %) in the study group were interested in receiving the phone call. While *n* = 22 (10.7 %) participants dropped out before the call (e.g., after M1 or after M3), *n* = 11 (5.3 %) declined the offer. Table 3 presents sample characteristics of those who declined and did not decline the call. When the call occurred, most participants (*n* = 88, 74.6 %) had already started working with the activity planner (M3). The call lasted for *M* = 13.16 min on average (*SD* = 6.99). In *n* = 56 cases (26.7 %), the phone call did not occur (e.g., due to scheduling conflicts or unavailability of the person). The participants still in the intervention who did not receive the phone call were sent the information via text via message on the platform.

**Table 3:** Characteristics study group.

Variables	Wanted call		Declined call
	Received it	Did not receive it	
*n*	118	56	11
Age in years (*M, SD*)	42.80, 13.01	41.64, 12.46	41.55, 13.26
Gender *n* (% female)	74 (62.7)	39 (70.9)	11 (100)
Education *n* (%)			
No certificate/lower secondary	1 (0.9)	2 (3.6)	0
Secondary school	17 (14.4)	12 (21.8)	1 (9.1)
Trade School	28 (23.7)	7 (12.7)	1 (9.1)
College	13 (11.0)	3 (5.5)	2 (18.2)
University	59 (50.0)	31 (56.4)	7 (63.6)
			
*Psychopathological symptoms (M, SD)*			
Depressive symptoms (BDI-II)	24.75, 6.32	25.31, 6.80	24.55, 6.46
Depressive symptoms (PHQ-9)	12.94, 3.37	11.95, 3.90	12.91, 4.01
Anxiety (GAD-7)	11.08, 3.80	10.73, 3.55	9.00, 4.80
Stress (PHQ-stress)	8.98, 3.42	9.66, 3.49	7.73, 5.04

*Note. n* = 185 (without *n* = 22 participants that dropped out before the call)*, M* = mean, *SD* = standard deviation; BDI-II = Beck's Depression inventory; GAD-7 = General Anxiety Disorder Screener; PHQ-9 = Patient Health Questionnaire-9; PHQ-stress = Patient Health Questionnaire Stress Module.

### 3.4 Treatment dropout rates

The pooled overall dropout rate across groups was 33.3 % (*n* = 131). Pooled dropout rates in the study group were 30.5 % (*n* = 63) and 36.1 % (*n* = 68) in the control group. Table 4 summarizes between-group effects. The odds ratios ranged between OR = 1.25 and 1.33, and the relative risks varied between RR = 1.08 and 1.10. Thus, the “risk” of completing the intervention was higher (about 9 % higher) in the study group. However, all confidence intervals overlap zero, indicating that the effects of offering an additional phone call on treatment dropout were non-significant (Table 4).

**Table 4:** Estimated effect of offering phone call on dropout (main analysis ITT).

Estimand	Analysis method	Estimated effect				
		OR	95 % CI	RR	95 % CI	RD
Unconditional	Unadjusted	1.33	(0.84, 2.10)	1.10	(0.94, 1.28)	–
Conditional	Adjusted	1.27	(0.76, 2.12)	–	–	–
Unconditional	Adjusted	1.25	(0.78, 1.99)	1.08	(0.92, 1.27)	0.06

*Note*. OR = Odd's Ratio, RR = Relative Risk, RD = Risk Difference, CI = Confidence Interval.

The sensitivity analysis targeting the hypothetical scenario (imputing dropout status of *n =* 56 individuals who wanted the additional phone call but did not receive it, sensitivity analysis 1) found a slightly larger odds ratio (range: OR = 1.40–1.46) and slightly larger relative risks (range: RR = 1.11–1.13) compared to the main analysis (see Table 5). However, all effects were non-significant as well. Except for the unconditional model, the effect estimates for setting all censored data points to “dropout” (sensitivity analysis 2) were similar and non-significant (see Table 5). For comparison, we also conducted a complete case analysis that yielded similar results and no significant effects (see Table 5).

**Table 5:** Estimated effect of offering phone call on dropout.

Estimand	Analysis method	Estimated effect			
		OR	95 % CI	RR	95 % CI
Unconditional	Unadjusted				
	Main Analysis	1.33	(0.84, 2.10)	1.10	(0.94, 1.28)
	Sensitivity Analysis 1	1.46	(0.90, 2.37)	1.13	(0.97, 1.32)
	Sensitivity Analysis 2	1.55	(1.04, 2.32)	1.14	(1.01, 1.25)
	Complete Case Analysis	1.31	(0.85, 2.01)	1.09	(0.94, 1.21)

Conditional	Adjusted				
	Main Analysis	1.27	(0.76, 2.12)	–	–
	Sensitivity Analysis 1	1.45	(0.85, 2.46)	–	–
	Sensitivity Analysis 2	1.51	(0.99, 2.27)	–	–
	Complete Case Analysis	1.23	(0.79, 1.93)	–	–

Unconditional	Adjusted				
	Main Analysis	1.25	(0.78, 1.99)	1.08	(0.92, 1.27)
	Sensitivity Analysis 1	1.40	(0.86, 2.26)	1.11	(0.95, 1.30)
	Sensitivity Analysis 2	1.48	(0.99, 2.20)	1.13	(1.00, 1.24)
	Complete Case Analysis	1.22	(0.79, 1.90)	1.07	(0.91, 1.19)

*Note*. OR = Odd's Ratio, RR = Relative Risk, CI = Confidence Interval; Main Analysis: Intention-to-treat, multiple imputation for censored data, Sensitivity Analysis 1: multiple imputation for dropout variable for “wanted call but did not receive it”, Sensitivity Analysis 2: censored data coded as “dropped out”, Complete Case: complete case analysis.

## 4 Discussion

Our study examined whether offering a single additional brief phone contact reduces treatment dropout in an internet-based intervention (IBI) for depressive symptoms with written guidance. While our main analysis did not yield statistically significant results, we observed a 6 % risk difference in treatment dropout rates between the study and control groups, favoring the study group.

This finding is consistent with previous research on unguided IBIs employing multiple additional phone calls, such as those conducted by Mira et al. (2017) for depressive symptoms (4 % reduction in dropout rates), by Levin et al. (2021) for distressed college students (6 % reduction), and Andersson et al. (2003) for recurrent headaches (3 % reduction). At the same time, our estimates are much smaller than the 18 % reduction in dropout rates reported by Pihlaja et al. (2020) caused by additional guidance in a guided IBI for depressive symptoms, as well as the 9 % and 14 % reductions reported in unguided IBIs for individuals with depressive symptoms (Gilbody et al., 2017) and obsessive-compulsive disorder, respectively (Kenwright et al., 2005). Thus, additional guidance via phone can have an effect on reducing dropout rates. Nevertheless, there is considerable heterogeneity between these study results, which several moderating variables may account for.

First, the studies employ different intensities of telephone-based support. While all other studies investigated multiple additional phone contacts, our study focused on a single phone contact. Consequently, our trial had substantially less phone-based contact time during the treatment than other studies with larger effects. For instance, in our study, the mean contact time was 13 min per patient, whereas Pihlaja et al. (2020) reported mean contact times of *M* = 73 min per patient over eight calls in total and Kenwright et al. (2005) reported mean contact times of *M* = 232 min per patient over nine calls in total. Understanding the right dosage is especially important in consideration of efficient resource allocation. Further studies should therefore examine how much additional support is required to reduce dropout rates significantly.

Secondly, the three studies reporting significantly larger effects also report significantly higher baseline rates of non-completion. In our study, 30 % of the study group and 37 % of the control group dropped out. The non-completion rates were higher in Pihlaja et al. (2020) (76 % in the study group vs. 94 % in the control group), Gilbody et al. (2017) (81 % in the study group vs. 90 % in the control group), and Kenwright et al. (2005) (50 % in the study group vs. 64 % in the control group). Similarly to our study, the studies that did not report significant effects also had relatively low overall dropout rates (18 % in the study group vs. 22 % in the control group, Mira et al. (2017); 29 % in the study group vs. 35 % in the control group, Andersson et al. (2003); 10 % in the study group vs. 13 % in the control group, Levin et al. (2021)). These findings suggest that when baseline dropout rates are already low, further improvements in adherence might be more challenging to achieve.

Additionally, all participants in our study received an initial call to conduct a structured diagnostic interview. These interviews usually lasted between 45 and 60 min. A meta-analysis demonstrated that contact before an IBI for depressive symptoms had a significant positive effect on treatment dropout compared to no contact (Krieger et al., 2023). None of the other studies of IBIs for depressive symptoms, included such an initial contact, as participants were recruited directly from primary care after receiving a diagnosis of depression (Gilbody et al., 2017; Pihlaja et al., 2020). It is not yet completely understood, how the initial contact is associated with treatment dropout rates. However, it might be another possible reason for the difficulty in detecting a significant additional effect of the brief phone call on treatment dropout, as the effect of personal contact might already have been implemented.

Assignment to study and control group was random. While random assignment is a strength, it may have masked an effect on participants at-risk for dropping out, as shown by Pihlaja et al. (2020). Future studies could benefit from focusing on at-risk participants. Therefore, understanding relevant predictors of treatment dropout is important to define meaningful criteria for at-risk participants in IBI for depressive symptoms. For example, participants who do not experience early improvement might be at higher risk of dropping out (Lutz et al., 2017). Moreover, a meta-analysis identified sex, age, level of education, anxiety, depressive symptoms, and stress as risk factors for dropout in unguided IBIs for depression (Karyotaki et al., 2015). No such systematic overview exists for guided IBIs for depression. This gap in the literature is worth exploring, as this information could be used to inform prediction models.

Lastly, our studies suggest that the participants' preferences should be considered. In our trial, a small proportion of individuals (mostly female, at least descriptively lower levels of stress and anxiety) declined to be called. Although the reasons are unknown, some individuals may choose an IBI specifically for its limited human interaction. Increasing the contact intensity could deter these participants. In a qualitative study on an IBI for procrastination, participants reported that increased therapist support could be experienced as aversive (Rozental et al., 2015). Given that no such evidence exists for IBIs for depressive symptoms, this is an important area for future research.

### 4.1 Strengths and limitations

We conducted the study using an effective and well-established IBI with well-trained and licensed psychotherapists as counselors. All counselors underwent specific training to ensure consistency in the delivery of the experimental intervention. At the same time, we explored the effect of offering additional contact in quite realistic settings, which includes the fact that not all individuals want an assigned intervention, and not all participants take part. Concurrently, our comprehensive sensitivity analysis demonstrated that the effects are quite robust against the assumptions we made.

However, some limitations should be considered. First, we cannot rule out that efficacy varies across counselors. With only eight counselors involved, the sample size was insufficient for a robust multilevel model (Bryan and Jenkins, 2015). However, we provided each counselor with mandatory training on the proper conduct during the additional phone calls and supplemented the training with a detailed manual to decrease heterogeneity. The study team encouraged counselors to contact them at any time, regarding any issues. Second, there were some issues in scheduling the additional contact. Due to the reliance on the existing IBI features, the contact had to be scheduled via a data-secure messaging system. This proved inconvenient for counselors and participants. We cannot rule out that providing a more convenient method for scheduling appointments could improve adherence rates and reduce the potential frustration participants may experience when they do not receive the scheduled phone call.

Lastly, even though we recruited a comparably large sample (*N* = 369 (Gilbody et al., 2017), *N* = 136 (Levin et al., 2021), *N* = 100 (Pihlaja et al., 2020), *N* = 88 (Mira et al., 2017), *N* = 44 ((Andersson et al., 2003), *N* = 44 (Kenwright et al., 2005)), our study's power was limited. A post-hoc power analysis (unadjusted OR of 1.3, α = 0.05, *N* = 394) revealed a power of 65 %. Due to the pragmatic approach of our study design and the fixed recruitment period, we could not extend the recruitment phase to achieve a larger sample size. It would be crucial for future studies to test the study design with a larger sample size to improve power and detect smaller effects.

## 5 Conclusion

A single additional phone call was associated with a 6 % lower but non-significant reduction in overall dropout rates. As the field moves toward scalable solutions, understanding who will benefit from different interventions or strategies to reduce treatment dropout is necessary to effectively allocate relevant resources. More research is needed to determine how additional contact can reduce treatment dropout, with a particular focus on additional contact and its interaction with other treatment characteristics.

## Funding

The development of the internet-based intervention and the accompanying trial this study is based on, was funded by the German public health insurance company “Techniker Krankenkasse”. The funding body was not involved in the study design, data collection, data analysis, and interpretation of the data, or in the decision to submit the article for publication.

## Declaration of competing interest

All authors declare that they have no known competing financial interests or personal relationships that could have appeared to influence the work reported in this paper.

## Appendix A Supplementary data

Supplementary material Image 1
