# Supplementary material for: Is one additional phone call enough? - Effectiveness of additional human support to reduce dropout from an internet-based intervention for depressive symptoms: A randomized-controlled trial
Source: Internet Interv. 2025 Mar 11;40:100818. doi: 10.1016/j.invent.2025.100818 (PMC11952021; doi:10.1016/j.invent.2025.100818)
Supplement: Supplementary file 1 — Supplementary material [file mmc1.docx]

**Supplementary material**

1. **Study procedure**

**
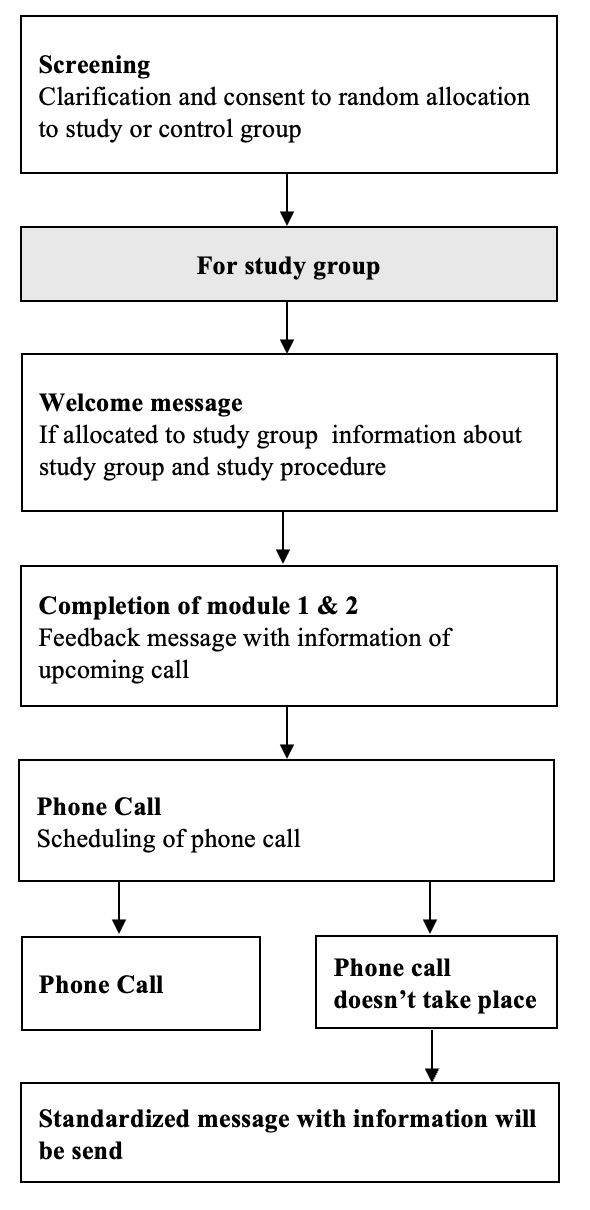
**

1. **Manual for study procedure in study group including phone guideline**

The complete study procedure in the study condition is described in detail below.

Welcome message

The welcome message is sent to all participants (study and control group). In the study group the following text element will be added to the welcome message.

Text element:

*[I would like you to benefit as much as possible from the coaching. In order to achieve this goal, I would like to support you with an additional telephone call of about 15 minutes after the second module. In this call, I would like to talk about the tasks that follow. I would like to give you a few tips on how you can make the best use of the modules in your everyday life. After the second module, I will suggest some time slots for this phone call].*

After module 1

There is also an additional text element for the feedback message after module 1.

Text element:

*[As already announced, I would like to contact you by telephone after the next writing assignment to talk about the further processing of the TC Depression Coach.*

*Please let me know when I can reach you for a 15-minute phone call. I would like to offer you the following time slots: [INSERT TIME SLOT]. The best thing to do is to work on the next writing assignment and add the time slot in which I can reach you directly in the writing assignment. If you are unable to arrange one of the specified time slots or need more time for the second writing assignment, please send me a separate message. Please also let me know if you don't want to talk on the phone - that's no problem either. I will then send you the additional tips as a message].*

ATTENTION: If a person does not respond regarding the appointment, the following short reminder message will be sent:

*Dear Ms. XX/Dear Mr. YY,*

*As announced in my last feedback, I would like to talk to you by telephone about your experiences with the coach. Unfortunately, you have not yet sent me any feedback on the proposed dates. If you are interested in this 15-minute telephone call, please let me know. If you don't want to talk on the phone, you can simply ignore this message and continue with your coaching as usual.*

After module 2

The following text element is available for the second feedback message to confirm the scheduled phone call.

Text element:

*[As discussed, I would now like to contact you by telephone to discuss the further processing of the TC DepressionCoach. You have indicated that you are [INSERT TIME WINDOW] easily reachable. I will therefore contact you on [INSERT CONCRETE DATE AND TIME WINDOW]*

After participants in the study group have completed module 2 and received their feedback, the phone call will take place.

*Hello Ms. XX/ Mr. YY,*

1. *Over the next few weeks, work in the Coach will change, as work with the daily planner will begin. I would like to talk to you briefly about this today. The daily planner is a very effective intervention. It can be particularly challenging to become active and anchor pleasant moments in your everyday life, especially with symptoms such as those you describe. To ensure that you start this task with the best possible support, it is important that we discuss the daily planner well in advance.*

*2a) Person has not yet started with day planner:*

*In the next week, the work in the coach will become more concrete. In particular, you will learn techniques that will help you to organize your activities in such a way that they have a positive effect on your well-being. The next modules will focus on working with a daily planner. As part of the task, you will learn how to give your day a more pleasant structure and plan activities that are good for you. Have you already read the task? (How did you understand the task and what were your initial thoughts on it?) [correct if necessary or briefly explain the most important aspects of the daily planner]. Our experience shows that minor difficulties can always arise when working with the daily planner. The daily planner is less about meticulously planning the day. It is particularly important that you consciously try to plan time for activities that are good for you and that make you feel good. This is where the daily planner in the TK DepressionCoach differs from the one you know from your everyday life. Is there something you like to do? Then your daily planner could look like this, for example: [Formulate an example that can ideally be derived from the client's goals]*

*I would like to give you a few specific tips:*

***Start small****: Start with small activities and gradually increase.*

***Follow the plan, not the whim****: Plan the activities as specifically as possible and carry them out at the specified times.*

*(****Just give it a try****: Just try activity planning as an experiment).*

***Don't get discouraged****: Even if you have not completed an activity, you have become active and taken the first step.*

*And very important:* ***reward yourself*** *when you have completed an activity.*

*2b) Person has already started using the day planner:*

*You are now already starting to work with the daily planner and have used it to learn a technique that can help you organize activities in a way that contributes to feeling good. How have you found it so far? [When person criticizes the daily planner: Correct perception, focus on XYZ]. Our experience shows that minor difficulties can always occur when working with the daily planner. How has this worked out for you so far? [brief feedback on this]. Once again the tip: The daily planner is less about planning the day meticulously. It is particularly important that you try to consciously plan time for activities that are good for you and have a positive effect on your well-being. This is where the daily planner differs from the calendar you may be familiar with from your everyday life. [If necessary, formulate an example here: "Is there something you like to do? Then your daily planner could look like this, for example: Formulate example"]*

*I would like to give you a few specific tips:*

***Start small****: Start with small activities and gradually increase.*

***Follow the plan, not the whim****: Plan the activities as specifically as possible and carry them out at the specified times.*

*(****Just give it a try****: Just try activity planning as an experiment).*

***Don't get discouraged****: Even if you have not completed an activity, you have become active and taken the first step.*

*And very important:* ***reward yourself*** *when you have completed an activity.*

*3) Conclusion:*

*Now you know what the work with the daily planner will look like in the coming weeks. [short summary of goals/what participants want to achieve/have set themselves]. Over the next few weeks, we will continue to work together to improve your well-being. This brings us to the end of the conversation and we will hear from each other again in the coach.*

**Dos and don'ts during the call**: In general, the phone calls for the study about the daily planner can be combined with other planned calls (e.g. if a client should be called due to worsening symptoms). In this case, it is only important to clearly separate the two topics in the call and to adjust the planned length of the call accordingly when coordinating the appointment. It is also important that the additional topics discussed are documented. To keep to the planned call duration of around 15 minutes, it is important to clearly state the aim of the call at the beginning. Generally, the writing assignments from module 1 and 2 should not be discussed again, as the participants have already received feedback in the second feedback letter. If individual aspects are to be addressed again, please note that the calls may take longer.

ATTENTION: If the person has not been reached, has not responded to the request to make an appointment, or has declined the call, the following message will be sent:

**Message**: Person has not yet started the day planner:

*Dear Ms. XX/ Dear Mr. YY,*

*Next week, the work in the coach will become even more concrete.*

*In particular, you will learn techniques that will help you to organize your activities in such a way that they have a positive effect on your well-being. The next modules will focus on working with a daily planner. My experience shows that there are always minor difficulties with this. As part of the task, you will learn to give your day a more pleasant structure and plan activities that are good for you. You may be familiar with keeping a calendar from your everyday life.*

*However, the daily planner is less about meticulously planning your day. It is particularly important that you try to consciously plan time for activities that are good for you and have a positive effect on your well-being. This is where the daily planner differs from the one you may be familiar with from your everyday life, which you primarily use to fulfill obligations.*

*So think carefully: are there things that you enjoy doing? You can then make time for these things in your daily planner.*

*I would like to give you a few specific tips:*

***Start small****: Start with small activities and gradually increase.*

***Follow the plan, not the whim****: Plan the activities as specifically as possible and carry them out at the specified times.*

*(****Just give it a try****: Just try activity planning as an experiment).*

***Don't get discouraged****: Even if you have not completed an activity, you have become active and taken the first step.*

*And very important:* ***reward yourself*** *when you have completed an activity.*

*[Best wishes*

*XX]*

**Message**: Person has already started the day planner:

*Dear Ms. XX/Dear Mr. YY,*

*I see you have already started using the day planner – that’s great! [ALTERNATIVE: You have already looked at the daily planner – that’s great!] You have become familiar with a technique that can help you to organize activities in such a way that they have a positive effect on your well-being. My experience shows that minor difficulties can always arise. So here's another tip: the daily planner is less about planning your day in minute detail. It is particularly important that you try to consciously plan time for activities that are good for you and have a positive effect on your well-being. This is where the daily planner differs from the one you know from your everyday life.*

*I would like to give you a few specific tips:*

***Start small****: Start with small activities and gradually increase.*

***Follow the plan, not the whim****: Plan the activities as specifically as possible and carry them out at the specified times.*

*(****Just give it a try****: Just try activity planning as an experiment).*

***Don't get discouraged****: Even if you have not completed an activity, you have become active and taken the first step.*

*And very important:* ***reward yourself*** *when you have completed an activity.*

*[Best wishes*

*XX]*
